# Dual-modality perfusion mapping in a complex pretibial wound: A case report

**DOI:** 10.1016/j.tcr.2026.101434

**Published:** 2026-09-15

**Authors:** Patrick James (P.J.) O'Donoghue, R. Pollock, J. Mockute, R. Dolan

**Affiliations:** a Department of Plastic & Reconstructive Surgery, St. Vincent's University Hospital, Dublin 4, Ireland; b UCD Conway Institute of Biomolecular and Biomedical Research, University College Dublin, Dublin, Ireland; c Royal College of Surgeons In Ireland (RCSI), Dublin, Ireland

**Keywords:** Pretibial haematoma, Pretibial laceration, Lower limb trauma, Infrared thermography, Indocyanine green fluorescence angiography, Perfusion mapping, Elderly patient

## Abstract

Complex pretibial wounds in older adults are common, difficult to clinically assess and at high risk of progression to skin necrosis. Despite this challenge, intra-operative assessment of viability remains subjective (colour, capillary refill and bleeding) and carries the risk of both under- and over-debridement. We describe a dual-modality approach combining handheld infrared thermography (IRT) and intra-operative indocyanine green fluorescence angiography (ICG-FA) to enable single-stage, precision debridement and resurfacing in a frail patient with a complex pretibial wound.

A 91-year-old man on apixaban presented with a pretibial haematoma/laceration following a road traffic collision. Bedside IRT using a handheld camera demonstrated a central hypothermic zone over the most contused skin. Following initial debridement guided by clinical assessment and IRT, ICG-FA performed with the SPY-PHI system (Stryker) demonstrated residual areas of reduced flourescence requiring further excision. Immediate split-thickness skin grafting and negative-pressure wound therapy were subsequently performed with uncomplicated recovery and complete graft take at first post-operative review. This combined strategy highlights the complementary roles of IRT for rapid, non-contact surface screening and ICG-FA for intra-operative perfusion assessment, together supporting accurate tissue preservation and immediate reconstruction.

## Highlights

• Dual-modality IRT + ICG-FA guided debridement in pretibial wound.
• Thermography identified a cooler zone prompting urgent operative management.
• ICG-FA revealed additional non-perfused skin beyond clinical judgement.
• Single-stage debridement with NPWT and STSG achieved complete healing.
• Workflow may aid decision-making in frail, anticoagulated pretibial trauma.

## Introduction

Pretibial soft-tissue injuries are common and occur predominantly in older adults [1]. Age-related dermal thinning, vascular disease and widespread anticoagulant use render the anterior tibial skin vulnerable to even minor trauma in the elderly. Clinically, assessment is subjective and variable and can contribute to both under and over-debridement of soft tissue [2]. Objective adjuncts may reduce this uncertainty. IRT detects gradients in skin-surface temperature that may correlate with perfusion and has been studied as a rapid, non-contact bedside tool in wound and burn assessment [3], [4]. In contrast, ICG-FA enables real-time visualisation of perfusion and has been reported as a useful clinical adjunct for guiding inta-operative decision making when viability is uncertain, however outcome data in limb trauma remain limited [5], [6], [7]. In this case, we suggest that a sequential IRT-to-ICG-FA workflow can optimise debridement accuracy and facilitate single-stage reconstruction in a frail patient with a complex pretibial wound.

## Case presentation

A 91-year-old man sustained a right pretibial soft-tissue injury following a low-velocity, single-vehicle road traffic collision (Fig. 1). He was anticoagulated with apixaban, which was withheld on admission. Radiographs confirmed no associated fracture, and the patient was managed according to Advanced Trauma Life Support (ATLS) protocol. Clinical assessment revealed a Dunkin grade III pretibial laceration requiring surgical debridement and reconstruction. The operative debridement strategy was performed as outlined below.

**Fig. 1:**
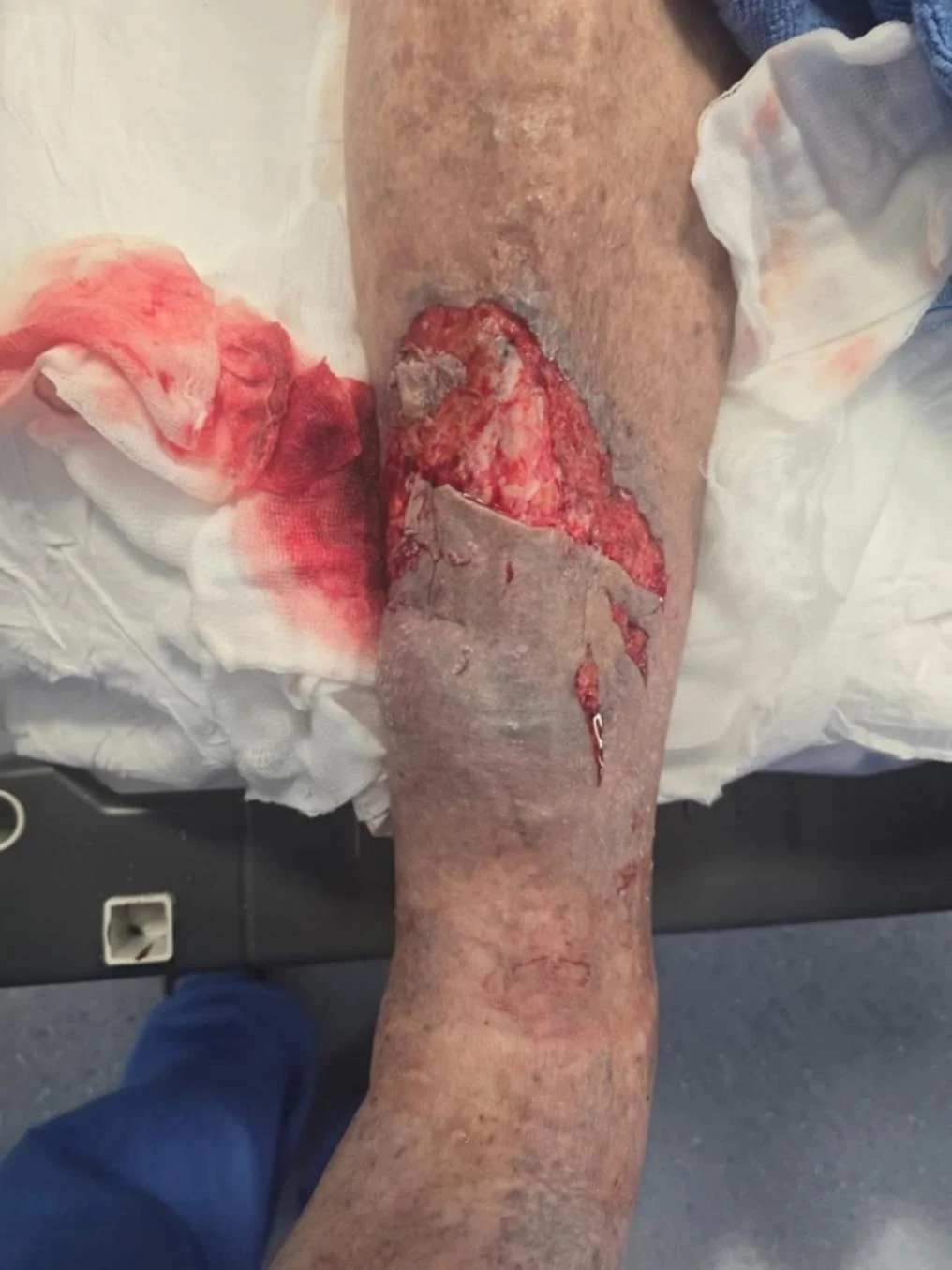
Preoperative clinical photograph of a Dunkin Grade III pretibial laceration in a frail patient.

**Step 1: Preoperative IRT**. A FLIR E5-XT handheld infrared camera was used perpendicular to the open skin surface at approx. 30 cm with attention to ambient control (draft minimisation, pre-exposure 15mins for thermal equilibrium). Default device emisivity was used. The thermal map demonstrated a central focal area of reduced surface temperature compared to adjacent skin (Fig. 2).

**Fig. 2:**
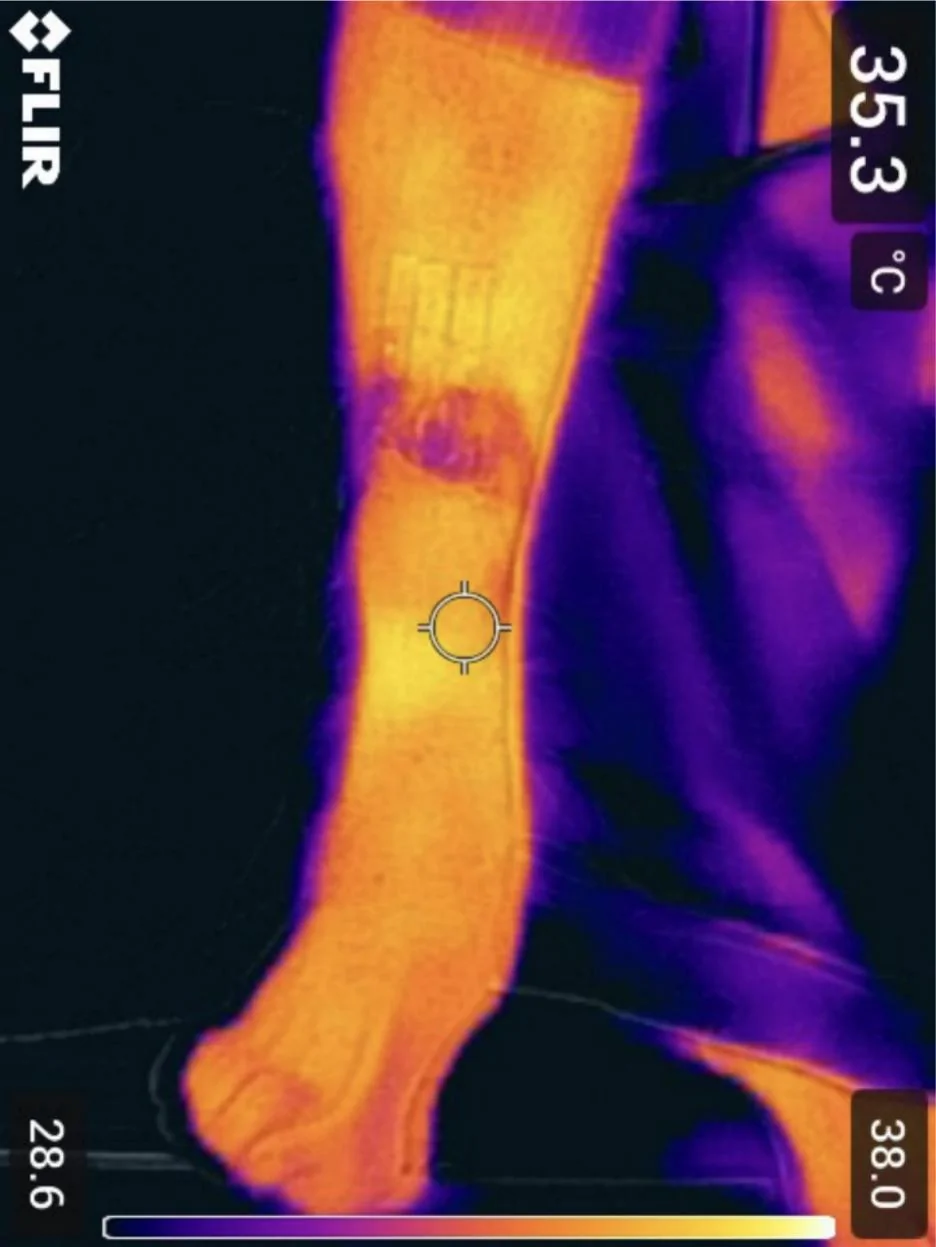
Pre-operative Infrared-Thermography (IRT) of the pre-tibial region showing area of decreased temperature.

**Step 2: Intra-operative ICG-FA**. Under spinal anaesthesia, initial debridement was performed using standard clinical indicators of viability before perfusion was objectively reassessed with ICG-FA. ICG-FA perfusion was assessed by fluorescence intensity and time-to-plateau relative to adjacent healthy skin. In line with published adjacent perfusion thresholds [8], regions demonstrating <25–33% relative perfusion compared with adjacent healthy skin were considered non-viable and underwent further debridement (Recognising not validated for pre-tibial trauma). Indocyanine green (ICG) 7.5 mg IV (3 mL of 2.5 mg/mL) was administered, followed by a 10 mL saline flush and imaged with the SPY-PHI system. Perfusion was assessed during the arterial inflow phase. This revealed 2 additional areas or markedly reduced flourescence that were not identified with IRT which prompted further debridement (Fig. 3).

**Fig. 3:**
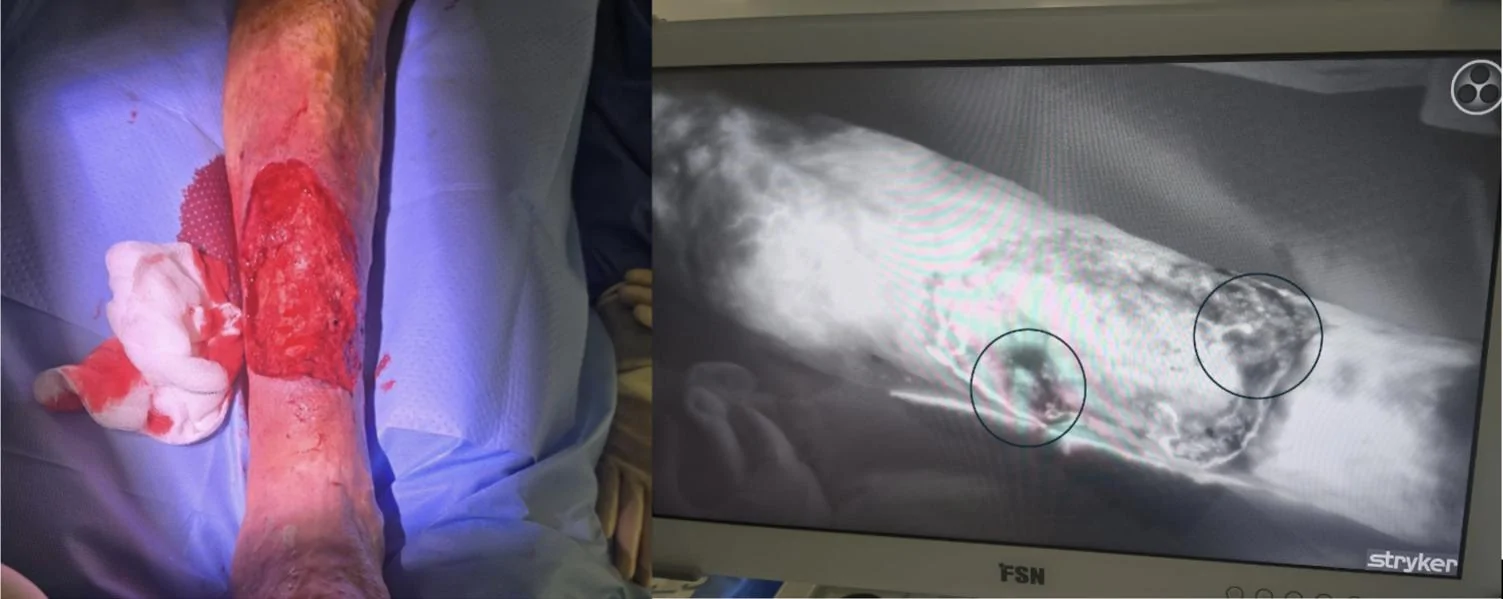
Intraoperative images post initial debridement and post ICG-FA with further circled devitalised areas.

**Step 3: Closure and Outcome**. The defect was resurfaced immediately with a split-thickness skin graft (0.010″, meshed 1.5:1), secured with staples and managed with negative pressure wound therapy at −125 mmHg over a silicone interface, as standard of care [9]. Graft take was 100% at day 6 post-operatively; by day 14 the patient was ambulating independently, with a stable, fully healed wound at 2-month follow-up (Fig. 4).

**Fig. 4:**
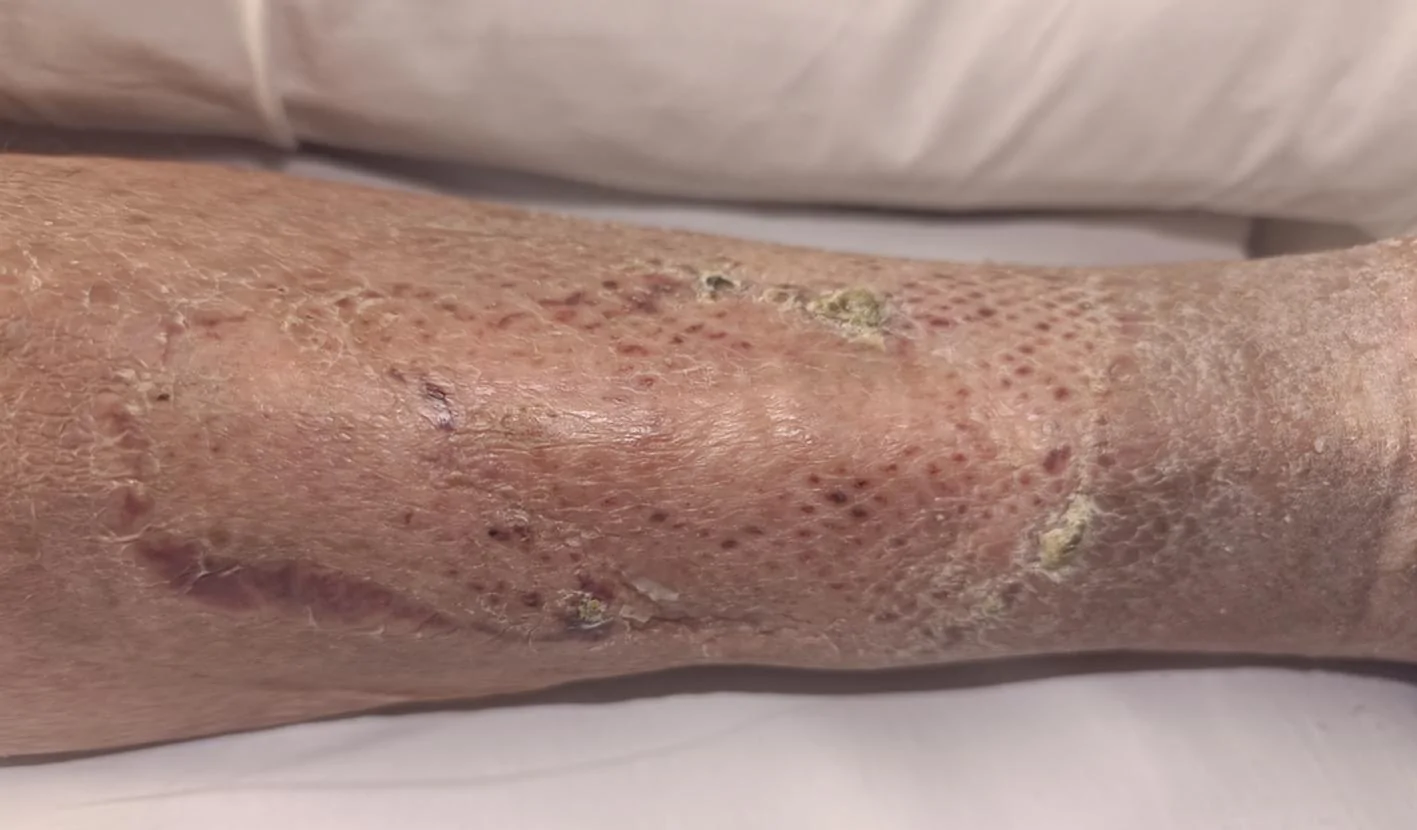
Clinical photograph of stable pretibial skin 2 months postoperatively.

## Discussion

Clinical relevance: Pretibial injuries in frail older adults are uniquely challenging, as soft-tissue viability can be difficult to judge clinically**.** In this case, a sequential dual-modality workflow combining infrared thermography (IRT) and indocyanine green fluorescence angiography (ICG-FA) enabled image-guided assessment of tissue perfusion and facilitated single-stage debridement and grafting. This approach may reduce the need for staged procedures by supporting earlier and more informed margin selection.

Complementary mechanism: IRT provides a non-invasive, rapid, surface assessment by detecting skin-temperature gradients that reflect underlying microvascular flow [3], [4]. It offers an immediate overview of at-risk areas that warrant closer inspection. ICG-FA, in contrast, directly visualises intravascular dye transit through the dermal and subdermal plexus, allowing real-time confirmation of perfusion and delineation of viable margins [5], [6], [7]. When used sequentially, these modalities offer complementary surface and depth information, optimising debridement precision and facilitating immediate reconstruction. However, it remains uncertain whether both modalities are required in all cases, and further comparative evaluation is needed to define the relative contribution and cost-effectiveness of each.

Comparison with other modalities: Techniques such as Laser Doppler Imaging (LDI) and near-infrared spectroscopy (NIRS) are described in the perfusion-assessment literature [3], [7] but are not routinely used intra-operatively due to equipment complexity, set-up time, and cost. Handheld IRT and portable ICG-FA systems offer practical advantages: rapid acquisition, ease of integration into existing workflows, and visual outputs that can be immediately interpreted by the operating team.

Implications and limitations: This combined workflow highlights the potential feasibility and impact of integrating IRT and ICG-FA into decision-making for complex soft-tissue wounds in co-morbid patients. However, this report is limited by single-case design, the absence of quantitative thresholds for IRT or ICG-FA and potential interpretation confounders.

## Conclusion

Dual-modality perfusion imaging (IRT + ICG-FA) may be a useful adjunct for early stratification and debridement margin selection in acute pretibial trauma, particularly in frail or anticoagulated patients and merits prospective evaluation.

## CRediT authorship contribution statement

**Patrick James (P.J.) O'Donoghue:** Methodology. **R. Pollock:** Writing – review & editing. **J. Mockute:** Resources. **R. Dolan:** Supervision, Conceptualization.

## Patient consent

Written informed consent for publication of this case and all images was obtained from the patient.

## Ethics statement

This report was deemed exempt from formal ethics approval.

## Funding

Funding in part by HSE Spark Seed Funding for equipment (SPY-PHI system).

## Declaration of competing interest

The authors declare no competing interests.

## Data Availability

No datasets were generated or analysed for this article.
